# Supporting the wounded: Parents of adolescents recovering from substance use disorder

**DOI:** 10.4102/hsag.v30i0.2787

**Published:** 2025-02-28

**Authors:** Faith Mathibela, Jan Masombuka

**Affiliations:** 1 Department of Social Work, Faculty of Social Sciences, University of South Africa, Pretoria, South Africa

**Keywords:** substance use disorder, substance, substance use, adolescents, parents

## Abstract

**Background:**

The increase of adolescent substance use disorders (SUDs) around the world has a lot of negative effects on the parents and frequently degrades their quality of life. Substance use disorder is a critical issue recognised as a chronic, complex health and social condition. Parents of adolescents recovering from SUD may suffer significantly as a result of the wide range of negative emotions that come with having adolescents recovering from SUD. From the extant literature, there is little evidence of the strategies used to address the support needs of parents who carry the burden of adolescent SUD care.

**Aim:**

The authors focused on exploring the support needs of parents living with adolescents recovering from SUD.

**Setting:**

The study was conducted in Tshwane, where they struggling with adolescent SUD issues, making it challenging to foster stable communities and social cohesion. Data were collected at the three in-patient treatment centres.

**Methods:**

Qualitative reseach was used to understand the phenomenon using ecological systems theory. The researchers conducted face to face interviews with parents, employing semi-structured method through purposive sampling.

**Results:**

The findings highlighted the following themes: The findings highlighted the following them: experiences of parenting an adolescent recovering from SUD, the support needed for parents and the desired services to help them cope.

**Conclusion:**

Parents expressed feeling overwhelmed and struggling to cope with adolescents recovering from SUD.

**Contribution:**

The study benefits the social work profession in the field of substance dependency by finding ways to support parents of adolescents recovering from SUD.

## Introduction

The global number of substance users rose to 275 million, while over 36m people were reported to be suffering from substance use disorders (SUDs), as indicated in the 2021 World Drug Report released by the United Nations Office on Drugs and Crime (UNODC) (2021). Adolescent SUD is a worldwide issue affecting adolescents in South Africa and across all racial groups (Masiko & Xinwa 2017). In Africa, recent research highlighted a high prevalence of SUDs among adolescents compared to the general population, with associated physical and psychosocial issues, including disrupted relationships with families and friends (Jumbe et al. 2021; Kaggwa et al. 2022; Mokwena & Setshego 2021). Substance use disorder is a widespread issue that has made it difficult for families to maintain their well-being, especially for the parents of adolescents with SUD (Hennessy, Cristello & Kelly 2019). Substance misuse is a global challenge that has placed an extensive challenge on the well-being of families, including the parents of adolescents misusing substances (Hlahla, Ngoatle & Mothiba 2023). Substance use disorder is a medical condition that is typified by notable deficits in health, social functioning, and voluntary control over substance abuse. Substance use disorder can result in health difficulties or challenges at work, school, or home (Danovitch & Mooney 2018). Olawole-Isaac et al. (2018) assert that SUDs have become more prevalent among adolescents in developing countries, particularly in Southern Africa. Approximately 7.6% of the South African population abuses substances, and one in every 14 people are regular users (Masiko & Xinnwa 2017).

Parents are devastated by the issue of adolescent SUD and require support because of the stress, frustration, and anger they experience concerning their adolescent’s substance misuse (Hlahla et al. 2023). However, for other reasons, the needs and concerns of the parents are mostly ignored and minimised. Parents of adolescents recovering from SUD may experience abandonment, anxiety, fear, anger, worry and embarrassment. At the same time, parents may wish to ignore or cut ties with the adolescents concerned (McCann, Polacsek & Lubman 2019). In extreme cases, parents may even feel they need to seek legal protection from the adolescent. Parents unable to regulate their adolescents’ behaviour may suffer catastrophic consequences because of their children misusing substances (Madiga & Mokwena, 2022).

The family is the fundamental unit of society, encompassing various definitions, and it holds significant importance in preventing the spread of SUD and combating it (Williams, Burton & Warzinski 2014). Parents are crucial in guiding adolescents to develop their strengths and resilience and achieve their full potential (Ðurišic & Bunijevac 2017; Masombuka 2021). Robust parental monitoring and open communication of values within the family can be a protective measure against substance dependence (Mathibela & Skhosana 2020). Furthermore, parents are vital in preventing and addressing their adolescents’ substance use; they can also help with interventions and support for recovery and sobriety (Williams et al. 2014).

Ecological systems theory was used to understand better the challenges faced by parents of adolescents recovering from SUD and how they can be supported to cope. Ecological systems theory indicates that the systems are interconnected and that each system’s influence depends upon its interactions with the others (Evans 2023). Ecological systems theory is centred on the fact that an individual is not an island; they are interlinked with various environmental systems: the microsystem, the mesosystem, the exosystem, the macrosystem, and the chronosystem (Ettekal & Mahoney 2017). From the aforementioned, it can be implied that adolescents recovering from SUD and their parents are subsystems that tie together a family as a system. Therefore, when an adolescent is using or recovering from SUD, the other parts of the family system become affected, and eventually, the entire system might collapse.

Despite the difficulty and burden of managing adolescent SUD, inadequate research focuses on parents as caregivers who assume responsibility for dealing with the problems associated with adolescents’ behaviour (Ngatweni 2018).

### Problem statement

A lot is being done to support adolescents with SUDs, but very little attention has been given to the support needs of parents (Groenewald & Bhana 2018). In other words, interventions have chiefly targeted individuals misusing substances and not the parents (McKeganey 2014). In addition to this, Choate (2015) highlights that social workers need to look into these needs and develop interventions that focus on addressing the support needs of parents. This is mainly because social workers are overwhelmed by work, and many lack the necessary tools or training to address substance-related issues. Based on the given information, the researcher noticed that there is limited information on how parents of adolescents recovering from SUD can be supported. This study aimed to gain an in-depth understanding of the challenges faced by parents of adolescents recovering from SUD and how they can be supported to cope.

## Research methods and designs

### Study design and setting

A qualitative research method was used to gain an in-depth understanding of the support needs of parents living with adolescents recovering from SUD. The study was conducted in Tshwane, where they are struggling with adolescent SUD issues, making it challenging to foster stable communities and social cohesion. Data were collected at the three in-patient treatment centres, which serve the social needs of substance users, including the needs of families whose problems emanate from adolescent SUD.

### Population and sampling

The population for this study comprised parents living with adolescents recovering from SUD. The researcher opted for purposive sampling because it allowed for selecting participants best suited for the study (Fouché, Strydom & Roestenburg 2021). The researchers conducted 15 interviews and found that the sample’s responses reached saturation, identified through the repetition of information. Participants were recruited from three in-patient treatment centres in the Tshwane region, explicitly catering for adolescents with the help of gatekeepers. To ensure the study was voluntary, prospective participants were given consent forms to sign if they agreed to participate. To ensure that only relevant data were collected, the researchers relied on the gatekeeper’s information, which was later verified through biological questions during data collection.

### Data collection

The researcher contacted potential participants to schedule appointments at social workers’ offices. The participants were informed about the study’s purpose and the data collection procedure. The main question asked was what are the support needs of parents with adolescents recovering from SUD. Furthermore, parents were asked how social workers could support them in the challenges they face while living with adolescents recovering from SUD.

Data were collected through semi-structured interviews using an interview guide to gather information. Semi-structured interviews helped the researcher obtain reliable and comparable qualitative data from parents of adolescents recovering from SUD (Kabir 2016). Probing and reflective techniques facilitated the researcher’s ability to pursue intriguing paths that arose during the interview sessions (Polit & Beck 2022). Data collection was concluded after interviewing the 15 participants, as data saturation had been reached.

Interviews were conducted in the social worker’s offices in English and Setswana to ensure a conducive environment and maintain confidentiality throughout the study. The duration of the interviews ranged between 45 min and 60 min, and field notes were also taken.

### Data analysis

The researchers transcribed the data and sought the assistance of an independent coder for data analysis. The researchers consulted with the independent coder to clarify the identified themes. Thematic analysis scrutinises the data collected to the point that themes emerge from the data. The thematic process comprises the following steps (Maguire & Delahunt 2017):

- *Step 1 – Familiarisation with the data*: Every audio-recorded interview was transcribed verbatim. Researcher 1 reviewed the data collected by reading the field notes taken during the interviews, listening to the recordings, and writing down all the information observed among the participants. The independent coder received the transcribed data and used the same data analysis procedure used by the researchers.
- *Step 2 – Coding*: The audio recordings were listened to multiple times, and took notes. Once satisfied with the recordings, the notes were sent to the transcriber for transcription. Afterwards, read the transcripts to fully comprehend the participants’ words.
- *Step 3 – Searching for themes*: Themes were generated from the data collected from the research participants using the coded data and associated texts. The list was then reduced by grouping related topics together to give meaning to the themes.
- *Step 4 – Reviewing themes*: The researcher, independent coder, and co-author collaborated to identify and merge themes that are similar or convey the same message.
- *Step 5 – Defining and naming themes*: The researcher and coder analysed each theme’s uniqueness and reached a consensus with the co-author on the identified themes. The researcher conducted an initial analysis and then discussed with the independent coder to reconcile the themes and categories identified independently by everyone. A further meeting with an independent coder was held to establish a consensus on the themes
- *Step 6 – Writing up*: Finally, the researcher started compiling the information as the research findings and integrating the storylines with the available literature.

### Measures of trustworthiness

A few specific research techniques are required to ensure trustworthiness of the study, namely credibility, transferebility, dependebility and conformability (Stahl & King 2020). Data verification is a procedure that permits the researcher to assess and verify the correctness and reliability of the information gathered following the completion of the data analysis process (Thomas 2011). The researcher utilised four pointers about reliability and validity to determine trustworthiness in this study: credibility, transferability, dependability, and conformability (Kumar 2014). The *credibility* of the research study was enriched through the triangulation of the data collection methods. Interpretations were discussed with the co-author to get confirmation. The researcher engaged more with the research participants to ensure she gathered comprehensive data about their experiences.

*Transferability* refers to the extent to which the study’s findings can be applied to other studies and contexts (Fouché et al. 2021), which was ensured by providing full details of the study’s settings, data-gathering processes for participants, data collection time and triangulation.

*Dependability* was improved by using peers, the transcriber, and an independent coder to analyse the data and offer feedback on the researcher’s reflexivity and any gaps in the procedures and data collecting. A semi-structured interview guide was used to record the interviews and collect data. The recording was given to the transcriber to transcribe the raw data collected. The researcher checked the transcriptions to ensure correctness.

The researcher used peers and participants to provide feedback on the findings and kept a reflective log of everything that happened in the field to ensure the findings’ *confirmability* or objectivity. The capacity to confirm themes in this study was made possible by the availability of raw data from the tape recorder and transcripts. Rebuilding data and synthesising developed concepts were also examined.

### Ethical considerations

The University of South Africa, College of Human Sciences Ethics Committee approved this study and issued an ethical clearance number (Rec-240816-052). The researchers also obtained permission from the Gauteng Department of Social Development (DSD) and the three in-patient centres, namely SANCA, Clear View Clinic, and Dr. Fabian and Florence Ribeiro Treatment Centre, to conduct the study. Participants were provided with details on the aim of the study, the anticipated time of their involvement, and the possible benefits and threats of participating in the study. After explaining the purpose and significance of the study to the participants, they were informed about confidentiality and were requested to sign consent forms should they agree to participate. The researcher debriefed participants immediately after each session to ensure that the emotions that surfaced during the interviews were addressed. This was done to minimise the emotional and psychological harm to participants.

## Results

The study participants comprised 15 parents of adolescents recovering from SUD, 11 females and 4 males. Four were self-employed hawkers and 7 worked full-time jobs. Parents are overwhelmingly occupied with work to cater to their family’s needs, which puts them at a disadvantage as their adolescent children remain unattended at home for extended periods. The biographical data on each participant’s gender, occupation and race, as well as the adolescents’ age and gender, are presented in Table 1.

**TABLE 1:** Biographical data of parents of adolescents recovering from SUD.

Participants	Gender	Age (years)	Relationship to the adolescent recovering from SUD	Number of times the adolescent was admitted for treatment
Participant 1	F	47	Mother	X2
Participant 2	F	45	Mother	X3
Participant 3	F	39	Mother	Lost count
Participant 4	F	43	Mother	X2
Participant 5	M	35	Father	X3
Participant 6	F	58	Mother	X3
Participant 7	F	54	Mother	X3
Participant 8	M	48	Father	X3, X1 Admitted to two different treatment centres
Participant 9	F	55	Mother	X2
Participant 10	F	42	Mother	X2
Participant 11	M	55	Father	X1, X3 Admitted to two different treatment centres
Participant 12	M	68	Grandpa	X2
Participant 13	F	48	Mother	X2
Participant 14	F	33	Mother	X1
Participant 15	F	42	Mother	X2

SUD, substance use disorder; F, female; M, male.

Of the 15 participants, 4 were fathers and 11 mothers. The researcher realised that based on gender distribution, more females are left responsible for caring for children. Mussida and Patimo (2020) support this statement, asserting that women are primarily responsible for caring for the home, the children, and other family duties. Therefore, the findings are also likely to be gender biased because of more information gathered from female participants.

The ages of the participants ranged from 33 years to 69 years. Seven participants were between the ages of 33 years and 45 years; 6 were aged between 46 years and 55 years; 2 were aged between 56 years and 68 years. The mid-30s to the mid-60s is considered middle adulthood, when an individual may meet their career goals, get involved with their hobbies, and explore other interests in their life (Fouché et al. 2021). At this stage, adults experience more intimately with others and explore more solid relationships; however, in the case of the participants interviewed in this study, they were experiencing more stress and health issues because of the stress of having an adolescent recovering from SUD.

Out of 15 participants, only four were married and stayed with their husbands. This indicates that most mothers are burdened with raising their adolescents alone. The research (Department of Statistics [Stats SA] 2018) suggests that the birth certificates of 61.7% of children born in South Africa did not include their fathers’ details. This implied that most fathers were not involved in the lives and upbringing of their children, leaving mothers to continue parenting alone. Mothers in this study also verbalised that the biological fathers failed to be there for their children after the divorce or separation from the mother.

Most of the adolescents recovering from SUD were admitted to a treatment centre more than once. According to Dykes and Casker (2021), the consequences of adolescents using substances do not only influence them but also affect their families, parents and society. The participants indicated that their adolescents are in the recovery process; however, most of the time, they relapse, putting more stress on the parents. The researchers realised that the high frequency of readmissions to treatment centres among adolescents in recovery indicates a significant burden on parents.

The discussion of themes and sub-themes generated from the interviews with parents living with adolescents recovering from SUD is presented in Table 2.

**TABLE 2:** Themes and sub-themes emenating from the parents of adolescents recovering from SUD.

Themes	Sub-themes
1. Parents’ accounts of their experiences in parenting an adolescent recovering from SUD	1.1 Parents found parenting difficult, frustrating and challenging 1.2 Parents voiced how they get stressed and find it difficult to trust an adolescent with SUD 1.3 Parents shared the financial constraints of living with an adolescent with SUD
2. Parents’ descriptions of the support they need as the parent of an adolescent recovering from SUD	2.1 Parents need more information 2.2 Parents need counselling and guidance
3. Parents’ descriptions of the support services they would like to receive as the parent of an adolescent recovering from SUD	3.1 Parents would like support groups for both parents and adolescents 3.2 Parents would like the community to be educated and community support mobilised

SUD, substance use disorder.

### Theme 1: Parents’ accounts of their experiences in parenting an adolescent recovering from substance use disorder

The challenges faced by parents of adolescents with SUD are constant in most aspects, and they frequently express stress, financial limitations, and coping difficulties in similar ways.

#### Sub-theme 1.1: Parents found parenting difficult, frustrating and challenging

Many parents recounted that parenting an adolescent recovering from a SUD is difficult, frustrating and challenging. Following are some of the frustrations that were verbalised by the parents who were interviewed:

‘It is frustrating to have a child who uses drugs. He has stopped using them now, but I do not know for how long! It is challenging; I have been trying very hard.’ (Participant 1, Female, 47 years)

‘[*I*]t’s frustrating having to monitor his movements. If we do not do that, he starts using they are back to using drugs.’ (Participant 3, Female, 39 years)

Kirst-Ashman and Hull (2015) argue that parents feel obligated to care for their children recovering from SUD, which is emotionally strenuous for them. The parent’s ability to manage the additional needs of adolescents recovering from SUD, maintain family responsibilities and employment commitments, and deal with unforeseen changes in their adolescents are among their other challenges (Hlahla et al. 2023). One of the parents interviewed had two adolescents who were both recovering from SUD.

#### Sub-theme 1.2: Parents voiced how they get stressed and find it difficult to trust an adolescent with substance use disorder

A good relationship and interaction between parents and adolescents are crucial for a healthy family. It is disturbing that parents shared that they find it difficult to trust their adolescents. This is indicated in the following narratives:

‘[*I*]t is not easy … mainly because recovery is not easy … because it is easy to get drugs. You can get it anywhere you want to, at school, at friends, at shops, anywhere you can think of you will find it … It is time-consuming because you must babysit them after they return from treatment.’ (Participant 4, Female, 58 years)

‘My life has changed; I do not feel comfortable anymore in my own house, I cannot trust my only son, I feel so frustrated and stressed all the time.’ (Participant 6, Female, 58 years)

Because of their stress levels, parents are excessively burdened and find it challenging to manage their daily lives. Shumway et al. (2019) assert that there is evidence that parents and other family members are having difficulty coping and suffering emotionally and psychologically because of adolescents with SUD. The fact that the parents could no longer trust their children, mainly regarding money, upset them. They felt they had no choice except to conceal money and other valuables inside the house.

#### Sub-theme 1.3: Parents shared the financial constraints of living with an adolescent with substance use disorder

Having an adolescent with SUD will directly impact friends and family members, especially financially and emotionally (Pons, Barron & Guijarro 2016). The parents of adolescents with SUD in this study mentioned that they are financially drained because of raising their adolescents with SUD. The following storylines support this statement:

‘[*L*]ast year alone, he has been to the SANCA in town two times, but when he gets home, he goes to the same group of friends and then ends up using drugs again. Financially, I cannot cope. It is too much money that I spend on his recovery.’ (Participant 1, Female, 47 years)

‘[*H*]e stayed there for three weeks, and then he looked so good. I am happy that he has changed, but now I have to pay continually for his trips to aftercare and buy test kits to test him regularly.’ (Participant 9, Female, 55 years)

Because of the expense of treatment at rehabilitation centres and overall maintenance of the adolescents, SUD also puts additional financial strain on parents (Groenewald & Bhana 2017). The responsibility for an adolescent with SUD also mainly lies on the parents, who must attend to family support, take time off work to participate in complaints from school, and provide financial assistance for the adolescent. Some of the parents expressed that in addition to managing the finances for their child’s treatment, they are also required to provide their child money to prevent them from stealing from their neighbours.

### Theme 2: Parents’ descriptions of the support they need as the parent of an adolescent recovering from substance use disorder

#### Sub-theme 2.1: Parents need more information

Most of the parents expressed the need for more information related to various aspects of their adolescent’s SUD and how to manage them. The following narrative speaks of the parents’ need for information:

‘I think getting information on what to look out for when your child has been discharged from the centre will work because we do not know how to handle them and what to do or react after treatment.’ (Participant 2, Female, 45 years)

‘I need more information on how to deal with him … What is the way forward, and when do I get my peace of mind? I need to know how to balance my life … without feeling guilty.’ (Participant 10, Female, 42 years)

As SUD is a chronic relapsing condition, parents need more support on how to continue supporting their adolescents as they continuously face the relapse challenge (Shumway et al.2019). Some parents feel left out in the process of getting information from professionals. It is alluded (Ngatweni 2018) that parents at times might feel sidetracked by counsellors in getting proper guidance and support on the knowledge of how to deal with adolescents recovering from SUD. Parents need to have sufficient information to talk with their children, especially adolescents recovering from SUD (Hlungwani et al. 2020). They also need information on how to take care of themselves and their mental health.

#### Sub-theme 2.2: Parents need counselling and guidance

Parents mentioned that they have not received adequate guidance on continuing and sustaining the support role to the adolescents recovering from a SUD. Parents verbalised that they need counselling or guidance to manage their adolescent’s recovery from a SUD and also counselling on how to cope:

‘I think I need to know how to communicate with the boys and how to help them. I need social workers to help us with information and guidance for getting help, especially for single parents like me.’ (Participant 8, Male, 48 years)

‘We need social workers to communicate more with us so that we can tell them what we are going through with these children in our homes.’ (Participant 9, Female, 55 years)

Parents continue to indicate that social workers expect a lot from them. The study by Hlahla et al. (2023) indicated that parents of adolescents recovering from SUD wanted more information and knowledge about SUD because they feel they cannot handle it. Research indicates that most programmes are lacking in supporting parents as they mainly focus on adolescents recovering from SUD (Baharudin & Sumari 2017).

### Theme 3: Parents’ descriptions of the support services they would like to receive as the parent of an adolescent recovering from substance use disorder

#### Sub-theme 3.1: Parents would like support groups for both parents and adolescents

The parents described how the support they would like to receive would be in the form of groups, and they mentioned groups for parents and groups for parents together with their adolescent children. The parents were clear that they would like to receive support by attending groups with other parents of adolescents recovering from SUD. About this sub-theme, the following was reported by the participants:

‘I would also appreciate having other parents in the same situation to talk to. Although it is very depressing to think about relapse, especially if the child has been clean for longer, it still happens.’ (Participant 6, Female, 58 years)

‘Support groups should be organised to allow parents to share their challenges and needs. Regular meetings with other parents are important as they will help us understand that we are not alone in this problem.’ (Participant 11, Male, 55 years)

Social workers have a specific role to play in providing parents with much-needed support and therapy services that are focused on supporting parents as their relationships that have become skewed as a result of adolescent SUD (Dykes & Casker 2021). Some of the parents also verbalised how having support groups will assist them in gaining more insights and a better understanding of the issue of adolescent SUD.

#### Sub-theme 3.2: Parents would like the community to be educated and community support mobilised

Many of the participating parents described how they would like the community to support them rather than judge them as parents of adolescents recovering from a SUD. They thought social workers could assist in this and specifically mentioned the churches. The following storylines describe the views of the parents:

‘I would also like to see community involvement because we will get better community support if everyone is involved. People do not understand what substance use is because even with us as their parents, we struggle to understand them.’ (Participant 3, Female, 39 years)

‘We would also love to be supported by our churches, but sometimes, the pastors don’t know how. Maybe social workers can train pastors to support parents because sometimes social workers are not available to assist.’ (Participant 15, Female, 42 years)

Parents believe that community members can be mobilised and be well-informed on the subject of SUD. They also shared that they felt the communities were ignoring the fact that if they did not work together in the fight against SUD, more problems would continue to affect them. A study by Masombuka (2021) supports this, stating that communities also need to be accountable for ensuring the safety of their children against substance use.

According to ecological systems theory, SUD impacts the individual, the family, and the community (Rogers, Gilbride & Dew 2018). Adolescent SUD is among the most highly stigmatised disorders, and some researchers consider SUD as a mental illness (Hogue et al. 2018). Some studies have identified stigma as a significant barrier for individuals to access available treatment services (Can & Tanriverdi 2015). To enhance knowledge and awareness among parents and adolescents, strategies to combat adolescents SUD should include family and community based programs to ensure a more effective response. In the fight against SUD and stigma, it is essential to mobilise community support and educate them to be in touch with the issues of SUD (Akdağa et al. 2018). Awareness campaigns should also address the challenges faced by parents of adolescents recovering from SUD.

## Discussion

The study aimed to explore the challenges faced by parents of adolescents recovering from SUD and their support needs to cope. From the collected data, it was evident that parents are deeply challenged and struggling to cope with adolescents recovering from SUD. The study confirmed that a lot is being done to support adolescents recovering from SUD; however, parents feel left out in the recovery process. Parents have expressed a need for support in coping with their adolescent child’s behaviour despite having gone through the treatment process. Trust remains an issue for parents because of everything they have been through, as some of them were victims of theft by adolescents (Masombuka & Mathibela 2022).

Regarding the challenges of difficulties in parenting, parents shared that they experienced negativity, especially as they also had to deal with stigma and challenges from community members. In support, Hlungwani et al. (2020) share that parents feel less motivated from time to time in dealing with adolescents recovering from SUD, especially when they keep on relapsing.

In relation to trusting the adolescent recovering from SUD, parents shared their frustrations as they cannot easily trust that the adolescent recovering from SUD has entirely changed. This is also indicated by McCann et al. (2019), stating that parents struggle to know what to do and what to avoid doing as their adolescent child recovers from SUD. Choate (2015) adds that in other situations, parents would find it tough and challenging to modify and accept the adolescent’s behaviour with SUD, which would make dealing with the problem more stressful.

In relation to financial constraints, some of the parents mentioned that they have to constantly miss work, which costs them money as they are mostly not at work because of attending issues relating to the adolescent with SUD. The pattern of adolescents recovering from SUD repeatedly going back to treatment centres is indicated to be one of the enormous financial burdens for parents as they constantly have to fund that. Groenewald and Bhana (2017) highlight that parents of adolescents with SUD face financial constraints as they have to attend family and school meetings and provide financial support.

Parents shared the need to get more information on SUD, recovery and relapses. Supporting the above statement, Masombuka (2021) shares that families need to be informed about how they can support adolescents abusing substances and recovering from SUD. Parents shared that they needed more information on dealing with adolescents recovering from SUD. In addition, there is a critical need for knowledge to comprehend what is happening with their adolescents’ lives and what has to be done moving forward (Hlahla et al. 2023).

In this regard, parents shared their need for counselling as they felt that they were struggling to cope with their situations. Choate (2015) states that, at times, the available intervention programmes are ineffective, leaving parents frustrated and dealing with adolescents recovering from SUD, not knowing where to go when they need to work on changing their lives. The parents also verbalised the need for social workers to be open and honest with them regarding their children’s recovery and how they can also be assisted in coping with their challenges. Parents need counselling as tensions between them and recovering adolescents have created huge problems, resulting in a lack of communication from both sides (Smith & Estefan 2014).

Regarding the support groups, parents indicated they need a platform to voice their frustrations and give their children the same platform to do likewise. In this regard, Ngatweni (2018) added that although parents need support to cope, they must also find better ways to understand their adolescents and continue supporting them. It indicates that although parents need support in coping with the recovering adolescent, they have not given up on their supporting role. Parents indicated that they believe their adolescent child will completely recover. A study by Masombuka and Mathibela (2022) revealed that parents needed social workers to provide parental skills to assist in empowering them to adopt better ways of building relationships with their recovering adolescents.

According to ecological systems theory, SUD impacts the individual, the family, and the community (Rogers et al. 2018). Adolescent SUD is among the most highly stigmatised disorders, and some researchers consider SUD as a mental illness (Hogue et al. 2018). Some studies have identified stigma as a significant barrier for individuals to come in and access available treatment services (Can & Tanriverdi 2015). In increasing the knowledge and awareness of adolescents, SUD strategies such as family and community-based programmes need to be utilised to guarantee a better response. In the fight against SUD and stigma, it is essential to mobilise community support and educate them to be in touch with the issues of SUD (Akdağa et al. 2018). Awareness campaigns should also address the challenges faced by parents of adolescents recovering from SUD.

### Limitations of the study

Parents of adolescents recovering from SUD were the only participants in this study, which took place in the City of Tshwane in the province of Gauteng. As a result, the findings cannot be generalised, but they can be used to support parents of adolescents recovering from SUD in similar contexts, such as other provinces. In addition, there was little diversity in language and ethnicity because the study only included parents of adolescents recovering from SUD in the three treatment centres based in the City of Tshwane region.

## Conclusion

The findings of this study indicate that parents need support in helping them cope with the challenges they face in raising and supporting adolescents who are recovering from SUD. Based on the parents’ challenges, it is concluded that more support systems must be implemented to ensure that parents are supported. It is also evident that other family members are affected, especially the siblings, because most of the focus is on the individual child who is recovering from SUD, and the other siblings have to take a back seat.

The findings also indicate that parents need assistance with parenting, coping and communication skills to help rebuild trust. By doing so, stronger family units will be reconstructed.

It is also concluded that support group sessions, which will include both the adolescents recovering from SUD and their parents, need to be encouraged to address the challenges faced by both parties in the recovery journey. The joint sessions will also assist parents in getting more information and understanding on the relapses and other issues related to SUD recovery, which will help ease their frustrations.

Social workers must acknowledge the central role of the parents, family, and community in dealing with SUD holistically (Appiah 2018). Finally, the authors also conclude that ecological systems theory can assist in holistically dealing with adolescent SUD, benefiting the whole family system.
